# Aerosolization Performance of Jet Nebulizers and Biopharmaceutical Aspects

**DOI:** 10.3390/pharmaceutics11080406

**Published:** 2019-08-11

**Authors:** Greta Adorni, Gerrit Seifert, Francesca Buttini, Gaia Colombo, Luciano A. Stecanella, Irene Krämer, Alessandra Rossi

**Affiliations:** 1 Food and Drug Department, University of Parma, Parco Area delle Scienze 27/A, 43124 Parma, Italy; 2 Apotheke, University Medical Center, Johannes Gutenberg University Mainz, Langenbeckstraße 1, 55131 Mainz, Germany; 3 Interdepartmental Center for Innovation in Health Products, BIOPHARMANET TEC, University of Parma, Parco Area delle Scienze 27/A, 43124 Parma, Italy; 4 Department of Life Sciences and Biotechnology, University of Ferrara, Via Fossato di Mortara 17/19, 44121 Ferrara, Italy

**Keywords:** nebulizers, aerosol output rate, aerosol output, mass median aerodynamic diameter, fine particle fraction, respirable dose delivery rate, respirable delivered dose

## Abstract

In this work, 13 jet nebulizers, some of which in different configurations, were investigated in order to identify the biopharmaceutical constraints related to the quality attributes of the medicinal products, which affect their safety, efficiency, compliance, and effectiveness. The aerosolization parameters, including the aerosol output, aerosol output rate, mass median aerodynamic diameter, and fine particle fraction, were determined according to the European Standard EN 13544-1, using sodium fluoride as a reference formulation. A comparison between the aerosol output nebulization time and the fine particle fraction displayed a correlation between the aerosol quality and the nebulization rate. Indeed, the quality of the nebulization significantly increased when the rate of aerosol emission was reduced. Moreover, the performance of the nebulizers was analyzed in terms of respirable delivered dose and respirable dose delivery rate, which characterize nebulization as the rate and amount of respirable product that could be deposited into the lungs. Depending on which of these two latter parameters was used, the nebulizers showed different performances. The differences, in terms of the rate and amount of delivered aerosol, could provide relevant information for the appropriate choice of nebulizer as a function of drug product, therapy, and patient characteristics.

## 1. Introduction

Nebulization is the oldest technique for the pulmonary administration of active substances using aerosol [1]. From the old vaporization devices to the recent full technology apparatuses, the objective still remains the generation of a proper aerosol and the deposition of an adequate drug dose in the lungs at the appropriate site and within a convenient time [2,3,4].

Nebulizers, as equipment (hardware), are primarily designed in terms of technical solutions, with the goal of assuring a predictable and reproducible aerosolization performance [4]. However, their use in drug therapy has important biopharmaceutical objectives. In the case of local or systemic drug activity, aerosol delivery and deposition on the lung epithelium leads to drug absorption. Therefore, nebulization products present in vitro characteristics related to their pharmaceutical development and in vivo properties linked to aerosol deposition and drug absorption [4,5]. Consequently, bioavailability parameters also have to be viewed as objectives of each drug product nebulization.

This in vitro and in vivo relationship reminds that inhalation products are the result of separated industrial competencies arising from a combination of the device and the formulated medicinal product. In the case of nebulization products, the combination consists of the nebulizer and the drug formulation. Both components account for efficacy by determining the availability (disposition) of the active substance [6,7,8,9,10,11]. The major barriers created by this combination derive from the fact that the manufacturers belong to different industrial sectors, i.e., the mechanical and the pharmaceutical industry [12,13,14]. They do not necessarily have the same product objectives. However, drug administration by nebulization merges these two different industrial competencies (i.e., know-how in formulation and the device) toward the final objective of the therapy’s success. For example, nebulization products for chronic and rare diseases are available on the market for coupling with a dedicated tested nebulizer [15,16,17,18]. In fact, variability in dose delivery from different nebulizers has been reported [8,19,20,21,22,23,24].

In this study, the pharmaceutical aspects of the nebulization process are explored to identify the biopharmaceutical constraints related to the quality attributes of medicinal products in order to assure their safety, efficiency, compliance, and effectiveness. The performance of a nebulizer in the inhalation of a drug solution or suspension was characterized by aerosol output (AO), aerosol output rate (AOR), mass median aerodynamic diameter (MMAD), geometric standard deviation (GSD), and fine particle fraction (FPF) parameters. The determination of these characteristics allows for a prediction of the amount of active substance that could be deposited in the respiratory tract [25].

In this work, the aerosolization performances of a previously selected number of jet nebulizers [26] were assessed by adopting various setting conditions and using sodium fluoride as a reference formulation, as indicated by EN 13544-1: 2007 + A1: 2009 [27]. The results were compared, and the biopharmaceutical significance of the measured parameters is discussed.

## 2. Materials and Methods

### 2.1. Materials

The materials used were sodium fluoride (NaF, ACS reagent ≥ 99%, batch MKBK1961V, Sigma Aldrich, Milan, Italy); total ionic strength adjustment buffer (TISAB) III solution (Merck KGaA, Darmstadt, Germany); a Type A/E glass filter 76 mm in diameter with a retention capacity of 99.98% (Pall Corporation, Port Washington, NY, USA) for the AO and AOR determinations; and Whatman Glass Microfiber filters 934-AH^TM^ 82.6 mm in diameter (GE Healthcare UK Limited, Buckinghamshire, UK) for the aerodynamic assessment.

The commercial jet nebulizers used in this study are summarized in Table 1. Each compressor was connected to an ampoule. In some cases, as reported in Table 1, the ampoule could be used in different configurations.

### 2.2. Methods

#### 2.2.1. Determination of AOR and AO

Aerosol output rate (AOR) and aerosol output (AO) values were determined according to the European Standard EN 13544-1: 2007 + A1: 2009. For the aspiration of the aerosol, a sine pump (Model SRU500CC, VCS, Parma, Italy) that reproduced the respiratory act through inhalation/exhalation was used. The volume of air moved was 500 mL with a cycle of 15 respiratory acts per minute and with a ratio of inhalation/exhalation equal to 1. The nebulization time for the AOR was 1 min, while the nebulization time for the AO was 1 min after the “sputtering”. An NaF solution in distilled water was prepared at a 1% (*w*/*v*) final concentration. The experiments were carried out in triplicate.

The determination of the AOR was performed as follows: the outlet of the nebulizer system equipped with a silicone rubber adapter was connected to the filter and its holder, and the latter to the sine pump. The ampoule was filled with 2 mL of the 1% (*w*/*v*) NaF solution (to be nebulized). The pump was switched on, and, 10 s later, so was the nebulizer. After 1 min, the nebulizer was switched off, and 5 s later, the sine pump was, too. The filter, the filter holder, and the dismountable connector from the outlet of the nebulizer system to the filter holder were dismantled. The amount of sodium fluoride in the components from the outlet of the nebulizer to the filter, included, was extracted and measured. The washing waters were transferred into 50-mL volumetric flasks containing 5 mL of TISAB III solution and were brought to volume with distilled water. Prior to this, the filter was wetted in the crystallizer with distilled water and sonicated for 5 min to favor NaF recovery.

The concentration of NaF in the volumetric flasks was determined in mV using an ion-selective electrode (Crison Strumenti S.p.A., Carpi, MO, Italy) connected to the potentiometer (pH meter Crison GPL21 S/N 145024 (Crison Instruments S.p.A., Carpi, MO, Italy)).

An analysis for the determination of the AO was carried out following the procedure described above, except for the nebulization time. The aerosol was collected in the filter inside the filter holder from the beginning of nebulization until one minute after the sputtering.

#### 2.2.2. Aerodynamic Assessment

The aerodynamic parameters (MMAD, GSD, and FPF) were determined according to the method described in the European Standard EN 13544-1: 2007 + A1: 2009. A next-generation impactor (NGI, Copley S/N NGI-0497 (Copley Scientific Limited, Nottingham, UK)) was used. The experiments were performed in triplicate.

The NGI was connected to an Erweka pump (model VP1000 S/N 11161406a7 (Erweka Italia S.R.L., Seveso, MB, Italy) via the solenoid valve of the critical flow controller (model TPK. Copley (Copley Scientific Limited, Nottingham, UK)). The NGI induction port was connected to the T-shaped glass tube using a silicone rubber adapter. A continuous aspiration flow of 15 L/min measured at the T-tube nebulizer port (Flowmeter Model DMF 2000; Copley Scientific Limited, Nottingham, UK) was applied for aerosol collection in the NGI. The mouthpiece of the ampoule, attached to the nebulizer, was connected to the T-shaped glass tube. The ampoule was filled with 2 mL of 2.5% (*w*/*v*) NaF solution. The nebulization time was fixed at 120 s, except for the nebulizers Pari Boy Sx (blue pisper) and Omron A3 Complete (position 1), in which it was 90 s and 45 s, respectively, due to a shorter sputtering time.

The Erweka pump and the TPK valve were switched on, and after 30 s, the nebulizer was activated. The aerosol was collected during the predetermined nebulization time in the NGI stages (1–7) and a micro-orifice collector (MOC), upon which a filter was placed. The nebulizer was switched off, and after 5 s, the Erweka pump and TPK valve were also switched off.

The ampoule, mouthpiece, silicone rubber adapters, T-shaped glass tube, induction port, NGI stages (1–7), and filter on the MOC were washed with distilled water, and the washing waters were transferred into 50-mL volumetric flasks each containing 5 mL of TISAB III solution and brought to volume with distilled water. Prior to this, the filter was wetted with distilled water in the crystallizer and sonicated for 5 min to favor NaF recovery. The concentration of NaF in the volumetric flasks was determined in mV using an ion-selective electrode connected to the potentiometer.

NaF standard solutions in the range of 10^−5^–10^−1^ mol/L were prepared for the construction of the calibration curve, which was used for the determination of fluoride with an ion-selective electrode.

#### 2.2.3. Data Processing

The values for the AOR, AO, MMAD, and FPF were determined using Excel and KaleidaGraph (Sinergy Software v.4.5.2, Sinergy Software, Reading, PA, USA).

## 3. Results and Discussion

According to the European Pharmacopoeia (Ph. Eur.), products intended for pulmonary administration by nebulization are tested with respect to the total amount of active substance delivered, the delivery rate, and the aerodynamic assessment of the nebulized aerosol [28]. These tests focus on the biopharmaceutical aspects of drug delivery. However, the same tests are used for the technical assessments of the nebulizer performance, typically regulated by the appropriate European Standard (whose code contains the letter EN). Since the products for nebulization require a combination of formulation and nebulizer, drug availability essentially depends on the formulation’s interaction with the selected nebulizer. It is not surprising that the same formulation is aerosolized differently in different nebulizers. The nebulizer’s performance is expressly regulated by the EN Standard on Respiratory Therapy Equipment [27]. The proposed use of NaF solution, as a surrogate for drug formulation, has the aim of standardizing the impact of the drug formulation on the nebulization performance. Thereby, the effect of the nebulizer on the aerosol delivery becomes apparent.

In characterizing the nebulization product, the drug emitted to the patient, measured as total output or drug delivered during a number of inhalation/exhalation cycles, is the quantitative parameter. The fine particle fraction is the qualitative parameter, i.e., the aerosol fraction having an aerodynamic diameter lower than 5 μm. Its measurement provides the aerosolized drug fraction at the size appropriate for deposition into the lungs. The third parameter, indicated by Ph. Eur. and EN, i.e., the output rate, is relative to the kinetics of nebulization: it is the amount of drug emitted in one minute of nebulization, usually the first minute.

The three nebulization parameters are decisive for in vivo drug disposition since they are bioavailability contributors. The respirable output obtained by multiplying the total output per the respirable fraction identifies the amount of drug potentially absorbable: it is reasonable to relate this parameter to a drug plasma profile measurable as systemic exposure (area under the curve (AUC)) [29]. It is known that the second determinant of bioequivalence (BE), i.e., the rate of absorption, derives from onsite drug dissolution and permeability. In the case of nebulization, the time of administration is generally shorter than 10 min. In oral drug administration, a dissolution time less than 15 min is considered to have no significant influence on BE.

Thus, the rate of absorption can barely be significantly affected by the time for aerosol deposition. Only in the case of prolonged release formulations, a slow drug release, and absorption could the plasma profile be affected; however, also in this case, the absorption rate’s dependence on the time of aerosol inhalation is improbable, since this time is quite short. In summary, the nebulization time has to be considered a marginal variable in the drug absorption rate.

**Figure 1:**
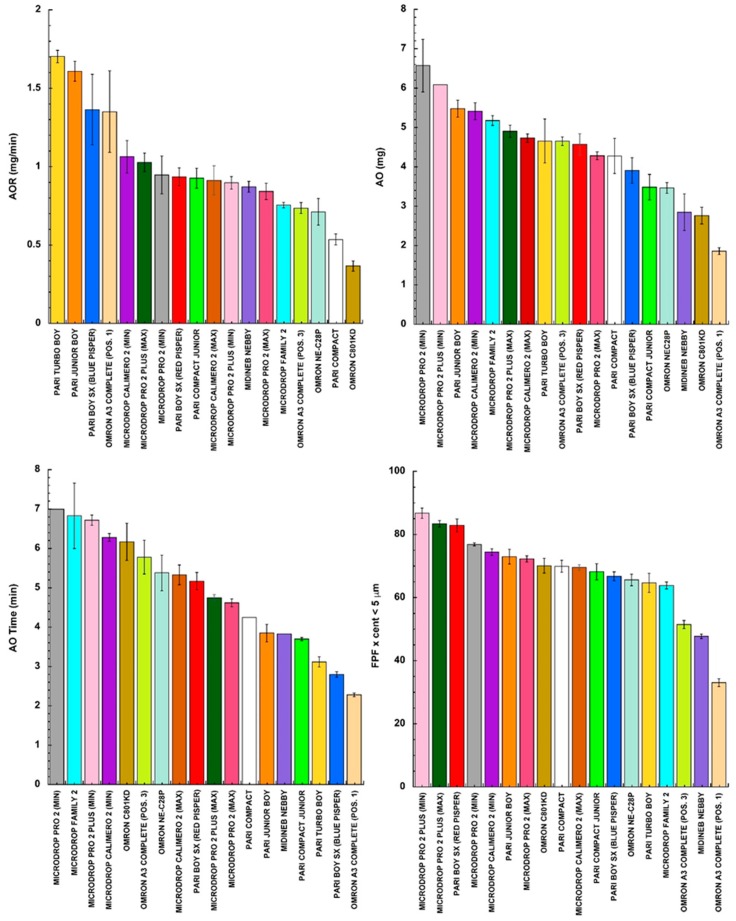
Aerosol output rate (AOR), aerosol output (AO), aerosolization time (AO time), and fine particle fraction (FPF) when 2 mL of NaF solution was aerosolized with the selected nebulizers from different manufacturers (mean value ± standard deviation, *n* = 3).

In Figure 1, the times required (AO time) to nebulize 2 mL of NaF solution are ranked for all of the tested jet nebulizers (operated according to the manufacturers’ instructions): some of them were used in different ampoule settings.

In the other panels of Figure 1, the measured values of the AOR, AO, and FPF are illustrated as well by ranking the values obtained in a descending order. Comparing the panels of the figure, the different nebulizers tested under the same conditions did not rank in the same order for all of the parameters considered.

The resulting MMADs are reported in Table 2. As the MMAD increased, the FPF decreased.

**Figure 2:**
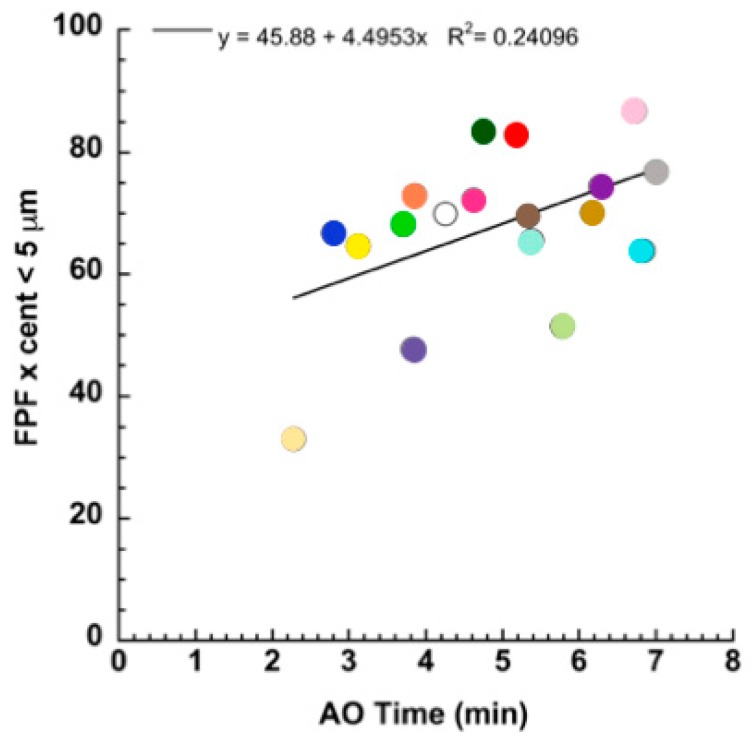
Relationship between the FPF and the aerosolization time.

In addition, through an attentive analysis of the data, one can see that the aerosol quality correlated with the nebulization rate. In fact, plotting the FPF versus the AO time, the quality of the nebulization significantly increased when the rate of aerosol emission decreased (Figure 2).

**Figure 3:**
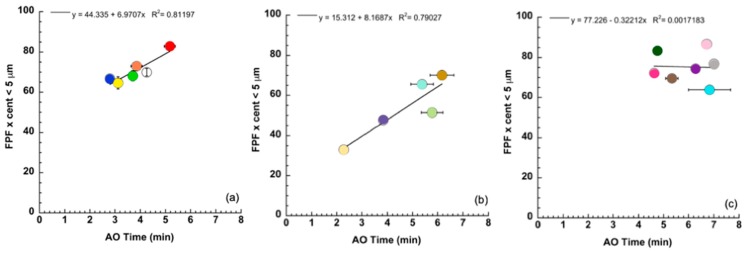
FPFs for the jet nebulizers manufactured by Pari (**a**), Omron (**b**), and Flaem (**c**) versus the time of nebulization (mean value ± standard deviation, *n* = 3).

The plot of Figure 2 shows the significance level of correlation considering the values collected from all of the nebulizers. The contribution of the individual nebulizer to this relationship could be evidenced by grouping the values exhibited by the nebulizers belonging to the same apparatus manufacturer. Figure 3 shows the FPF versus the time of nebulization for the apparatuses manufactured by Pari, Omron, and Flaem. A greater influence of the nebulization rate on the FPF for the Pari and Omron nebulizers than for the Flaem nebulizers was observed.

In summary, the nebulization rate of the same amount of solution aerosolized can have a significant effect on the respirability of the aerosol. In general, a decrease in the fine particle fraction was observed when the time of aerosolization was shortened. However, this negative effect can be counteracted by the technology developed in the nebulizer.

The EN parameters presented in Figure 1 are frequently combined in an attempt to compare the different nebulizers in a more comprehensive manner. For example, some authors have calculated the respirable dose delivery rate parameter (RDDR) together, multiplying the aerosol output rate by the fine particle fraction [30]. Other researchers have preferred to quantify the total respirable delivered dose (RDD) by multiplying the aerosol output by the FPF [8]. Substantially, these two combined parameters have different biopharmaceutical meanings, since the first is related to the deposition rate of respirable aerosol, whereas the second represents the amount of respirable aerosol deposited. Therefore, they differently evaluate the performance of a nebulizer, characterizing nebulization as the rate and amount of respirable product deposited into the lungs.

We have already anticipated that the rate of drug deposition during nebulization per se has to be considered slightly influential on the rate of bioavailability (absorption). The time interval of the inhalation administration is too short for highlighting differences in BE. More likely, this parameter characterizes the time of nebulization that is relevant to the patient’s convenience. Alternatively, the RDD relates to drug exposure, which in the case of drug absorption determines the area under the plasma profile.

**Figure 4:**
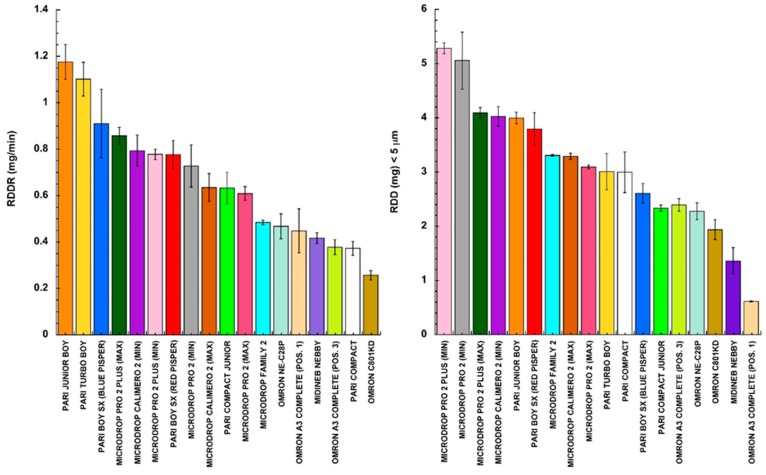
Respirable dose delivery rate (RDDR) and the total respirable delivered dose (RDD) ranked accordingly to the nebulizer used (mean value ± standard deviation, *n* = 3).

The combined parameters measuring the respirable dose delivery rate (RDDR) and the total respirable delivered dose (RDD) were calculated from the data reported in Figure 1. The values are plotted in the bar graphs of Figure 4 according to a decreasing rank order and using the same colors to identify the nebulizers.

Comparing the two bar graphs, evidence emerged that the various nebulizers ranked differently depending on their characteristics, which was evidenced by the combined parameter considered. This does not indicate that one nebulizer is better than another one, since each nebulizer works in combination with the specific formulation it delivers. The authors do not intend to generalize or claim superiority, since it is clear that the most significant parameter to take into account in nebulizer performance is strictly related to drug activity and formulation combined. The bar graphs only have the meaning of ranking the characteristics of jet nebulizer product depositions in terms of rate and “extent” of the respirable delivered aerosol, using a standard NaF solution as formulation.

## 4. Conclusions

The rate and extent of drug absorption from dosage forms are the determinants of drug bioequivalence. The nebulization time determines the total respirable dose deposited and, in the case of absorption, it is proportional to the AUC of the drug plasma profile. In the usual conditions of nebulizer usage, the rate of drug absorption after nebulization is marginally affected by the nebulization time. Due to patient convenience reasons, extending the nebulization time in order to control the absorption rate is not comparable to a drug release control after oral administration of a sustained release product.

The parameters RDDR or RDD, here presented for a large group of jet nebulizers, offer to healthcare providers relevant information on the appropriate use of apparatuses. The user can select the nebulizer characteristics depending on drug properties, therapy needs, patient respiration characteristics, and results needed. Drug activity and the therapeutic objective of nebulization directs the nebulizer choice for therapy effectiveness (provided by the rate and “extent” of nebulization).

Finally, it should be underlined that this study was conducted with a standard formulation, i.e., a sodium fluoride solution. Using an actual drug formulation, the performances could substantially change the results illustrated, since a nebulized aerosol is the result of a combination of an apparatus and a formulation.

## Figures and Tables

**Table 1:** List of the pneumatic nebulizers used in this study.

Nebulizer	Manufacturer	Batch (S/N)	Configuration	Identification Color	
Pari Compact	Pari	2W17C10078		White	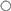
Pari Compact Junior	Pari	2W17A13163		Light green	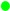
Pari Boy SX	Pari	2W17B01844	Blue Pisper *	Blue	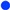
			Red Pisper *	Red	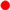
Pari JuniorBoy SX	Pari	2W17B08883		Orange	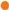
Pari TurboBoy SX	Pari	2W16H00598		Yellow	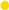
Microdrop Family 2	Flaem Nuova	16A 155 0873		Light blue	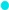
Microdrop Calimero 2	Flaem Nuova	16AF450652	Ampoule Valve MAX	Brown	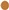
			Ampoule Valve MIN	Purple	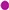
Microdrop Pro 2	Flaem Nuova	15 A7870439	Ampoule Valve MAX	Fuchsia	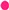
			Ampoule Valve MIN	Grey	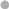
Microdrop Pro 2 Plus	Flaem Nuova	Engineering sample	Ampoule Valve MAX	Dark green	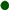
			Ampoule Valve MIN	Pink	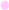
Omron C801KD	Omron Healthcare	20160600989VF		Yellow-green	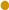
Omron NE-C28P	Omron Healthcare	20160905635UF		Green water	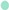
Omron A3 Complete	3A Healthcare	201702/00279F	Ampoule Position 1	Blush	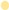
			Ampoule Position 3	Lemon green	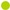
Midineb Nebby	3A Healthcare	16/30635		Lilac	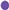

* nebulizer nozzle.

**Table 2:** MMAD (mass median aerodynamic diameter) and GSD (geometric standard deviation) (mean ± standard deviation, *n* = 3) resulting from nebulization of NaF solution with the selected nebulizers.

Nebulizer	Configuration	MMAD (μm)	GSD
Pari Compact		3.21 ± 0.15	2.27 ± 0.03
Pari Compact Junior		3.38 ± 0.19	2.22 ± 0.01
Pari Boy SX	Blue Pisper	3.48 ± 0.09	2.21 ± 0.03
	Red Pisper	2.56 ± 0.12	1.99 ± 0.02
Pari Junior Boy SX		3.14 ± 0.12	2.10 ± 0.04
Pari Turbo Boy SX		3.67 ± 0.20	2.19 ± 0.05
Microdrop Family 2		3.65 ± 0.07	2.12 ± 0.01
Microdrop Calimero 2	Ampoule Valve MAX	3.28 ± 0.09	2.19 ± 0.03
	Ampoule Valve MIN	2.90 ± 0.03	2.22 ± 0.05
Microdrop Pro 2	Ampoule Valve MAX	3.14 ± 0.06	2.10 ± 0.02
	Ampoule Valve MIN	2.84 ± 0.07	2.10 ± 0.05
Microdrop Pro 2 Plus	Ampoule Valve MAX	2.47 ± 0.09	2.02 ± 0.03
	Ampoule Valve MIN	2.14 ± 0.08	2.12 ± 0.02
Omron C801KD		3.25 ± 0.15	2.04 ± 0.02
Omron NE-C28P		3.63 ± 0.12	2.05 ± 0.03
Omron A3 Complete	Ampoule Position 1	6.76 ± 0.16	2.54 ± 0.02
	Ampoule Position 3	4.44 ± 0.06	2.12 ± 0.03
Midineb Nebby		4.97 ± 0.01	2.16 ± 0.03
